# Case Report: Asymmetric Bone Marrow Involvement in Patients With Acute Leukemia After Allogeneic Hematopoietic Stem Cell Transplantation

**DOI:** 10.3389/fonc.2021.626018

**Published:** 2021-03-04

**Authors:** Han Yan, Zhenyang Zhou, Yingying Wu, Zhaodong Zhong, Yong You, Junxia Yao, Wanxin Chen, Linghui Xia, Xiaotian Xia, Wei Shi

**Affiliations:** ^1^Institute of Hematology, Union Hospital, Tongji Medical College, Huazhong University of Science and Technology, Wuhan, China; ^2^Department of Radiology, Union Hospital, Tongji Medical College, Huazhong University of Science and Technology, Wuhan, China; ^3^Hubei Province Key Laboratory of Molecular Imaging, Wuhan, China; ^4^Department of Nuclear Medicine, Union Hospital, Tongji Medical College, Huazhong University of Science and Technology, Wuhan, China; ^5^Fred Hutchinson Cancer Research Center, Seattle, WA, United States

**Keywords:** asymmetric, blasts, relapse, acute leukemia, allogeneic hematopoietic stem cell transplantation, ^18^F-FDG-PET/CT, case report

## Abstract

After allogeneic hematopoietic stem cell transplantation (allo-HSCT), acute leukemia relapse is common, and asymmetric bone marrow recurrence hasn't been reported. Because the anatomical distribution of acute leukemia clones in the bone marrow after allo-HSCT is presumed to be diffuse, bone marrow aspirations are performed in single site. The first case was a 20-year-old man who was diagnosed with acute myelomonocytic leukemia and received haploidentical allo-HSCT. Routine bone marrow biopsy of his left posterior iliac bone marrow showed 52% leukemia blasts, while the right side had 0% blasts 10 days later. The second case was a 23-year-old woman who was diagnosed with acute B lymphoblastic leukemia and received HLA-identical sibling allo-HSCT. Although 62% of blasts were found in her left iliac marrow on day +122, 0% of blasts were found on a sample obtained from the right iliac crest on day +128. Bilateral iliac bone marrow pathology and whole-body ^18^F-FDG PET/CT scans confirmed that the leukemic infiltration in her bone marrow was asymmetric. To our knowledge, these are the first case reports of asymmetric bone marrow infiltration of blasts in acute leukemia patients after allo-HSCT. Bilateral posterior iliac crest aspirations or ^18^F-FDG-PET/CT scans may help distinguish such involvement.

## Introduction

Allogeneic hematopoietic stem cell transplantation (allo-HSCT) is a curative option for patients with acute leukemia. Post-HSCT recurrence still represents the major cause of treatment failure and up to 50% of acute leukemia patients will relapse (1). Periodic bone marrow aspiration is performed to monitor for early relapse (2). Although the anatomical distribution of acute leukemia clones after allo-HSCT has not been studied (3), blasts are generally considered to be uniformly infiltrated throughout the bone marrow system. Bone marrow aspirations are performed in one site for patients with acute leukemia (4). For patients with relapse or suspected relapse, aspirations may be conducted at 0.5 to 3-month intervals according to different therapy protocols or attending physician preference.

In serial aspirations, it is not uncommon to see inconsistent residual disease burden in different iliac crests. Differences are usually attributed to providers' operational errors, blood-diluted bone marrow, incorrect enumeration by pathologists, incorrect machine measurement, or variable graft vs. leukemia (GVL) response. However, the following two cases reveal a rare cause of inconsistent bone marrow aspiration results. All aspirations were performed on the posterior iliac crests according to published protocols and were conducted by the same providers (5).

## Manuscript Formatting

### Case Description

#### Case 1

In August 2015, a 20-year-old man was diagnosed as acute myelomonocytic leukemia. The cytogenetic risk stratification was intermediate. His bone marrow showed complete remission (CR) after two courses of induction. Following three courses of consolidation, granulocyte-colony stimulating factor (G-CSF)-mobilized peripheral blood stem cell (PBSC) from his human leukocyte antigen (HLA) 4/6 matched father was infused on April 20–21, 2016. The conditioning regimen consisted of decitabine (20 mg/m2/day, −11 to −7), cytarabine (3 g/m2/day, −9 to −7), busulfan (3.2 mg/kg/day, −5 and −4), cyclophosphamide (1.8 g/m2/day, −3 and −2). A regimen of tacrolimus, short-term methotrexate (MTX) and mycophenolate mofetil was given for graft vs. host disease (GVHD) prophylaxis. His neutrophils and platelets were engrafted on day +10 and +13, respectively. On day +30, his bone marrow showed CR and complete donor chimerism, and the results were normal until November 16, 2017. Because of repeated mild liver chronic GVHD, tacrolimus was not withdrawn until day +350. Despite no physical symptoms and normal peripheral blood counts, his left posterior iliac bone marrow aspirate smear revealed a blast percentage of 52% during a routine follow-up on Mar 7, 2018. The blasts expressed CD34 dim, CD117, CD33 strong, HLA-DR, CD13, and did not express CD7, CD19, CD56, CD11b. The immunophenotype was the same as the first diagnosis. He declined all treatment recommendations and insisted on repeating bone marrow aspiration as soon as possible. Ten days later, as shown in Table 1, sampling of the right posterior bone marrow aspirates not only showed a blast percentage of 0%, but also a recipient chimerism percentage of 0%. No pathologic genetic abnormalities or meaningful mutations were found. The patient and his parents declined further treatment and only agreed to reexamination after 2 weeks. Two weeks later, with normal peripheral blood cells, the blast percentage of his left posterior iliac bone marrow smear was still 52%. The blasts expressed the same immunophenotype as before. No blast forms were seen in the peripheral blood smear. Unfortunately, due to the inconsistent results, the patient declined further intervention and died of high leukocyte syndrome 4 months later.

**Table 1:** The aspiration results of case one during follow-up.

**NO**.	**Date**	**Morphology** ** (%blasts)**	**MFC** ** (%blasts)**	**STR** **(% recipient)**	**Status**
**Case 1**	April 20–21,2016				HSCT
	November 16, 2017	0	0	0	Right
	Mar 7, 2018	52	6.58	N	Left
	Mar 17, 2018	0	0	0	Right
	Mar 27, 2018	52	23.4	N	Left

*STR, short tandem repeat mismatches; N, no detection; Right, aspirates from the right posterior iliac crest; Left, aspirates from the left posterior iliac*.

#### Case 2

**Figure 1:**
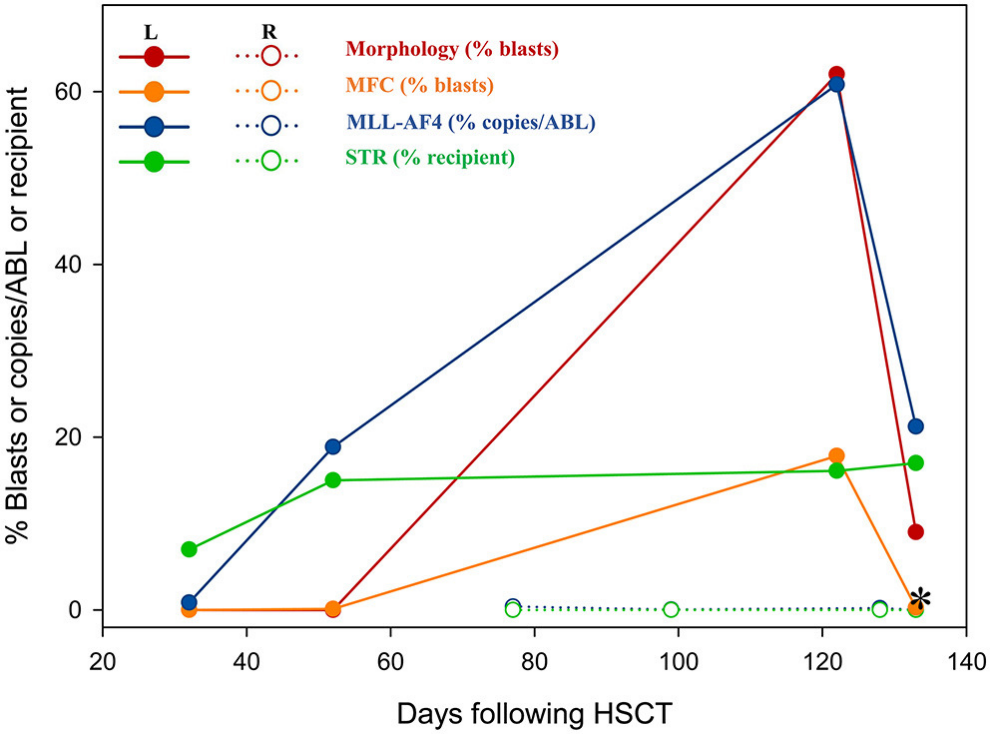
The residual disease was detected by morphology, MFC, RT-PCR and STR. *, the day +133 for a bilateral posterior iliac crest bone marrow biopsy. STR, short tandem repeat mismatches; R, aspirates from the right posterior iliac crest; L, aspirates from the left posterior iliac.

**Figure 2:**
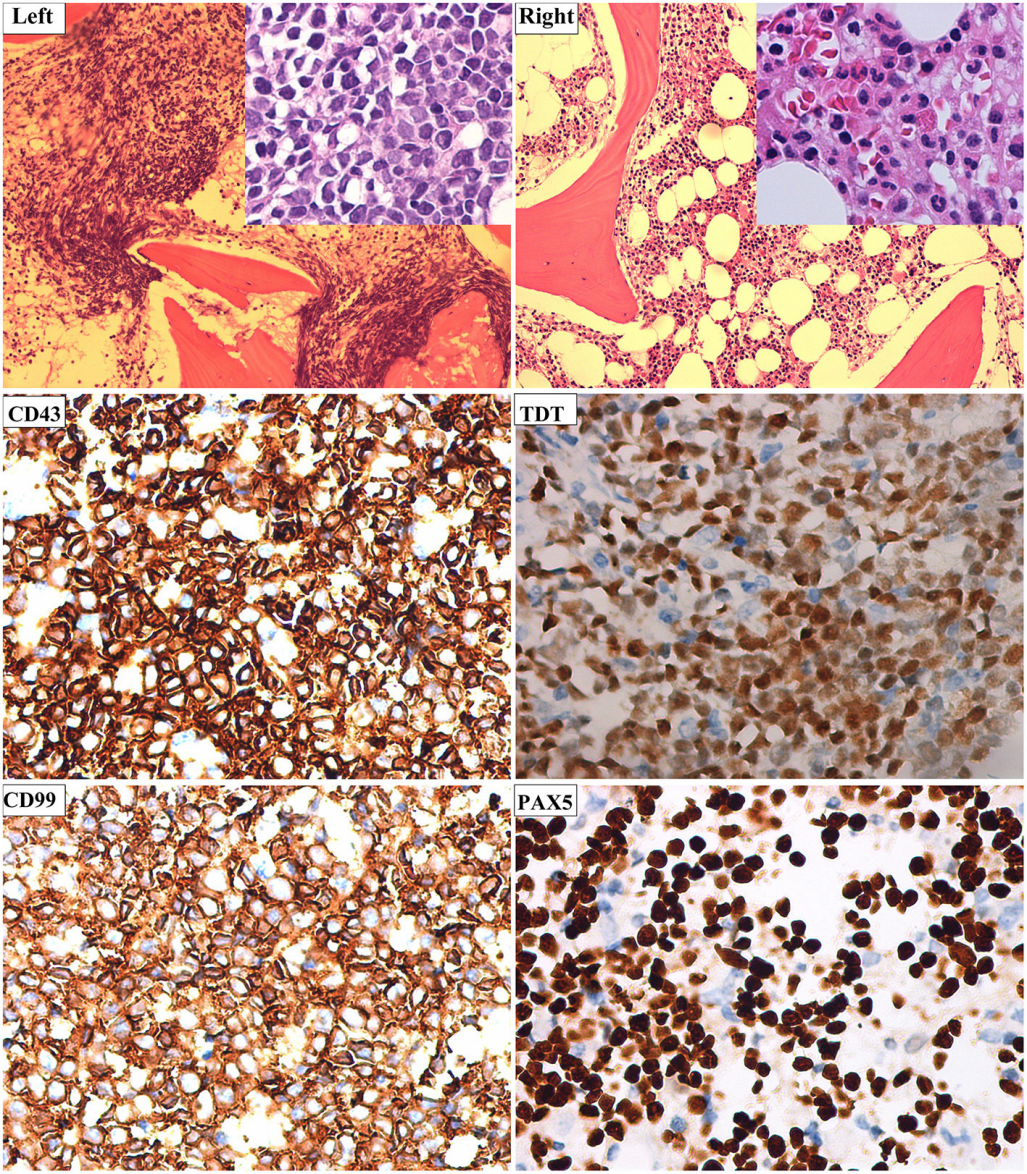
The bone marrow biopsy was performed in bilateral posterior iliac crests. **Left**, left posterior iliac bone marrow (hematoxylin and eosin, ×100) was filled with B lymphocyte leukemia cells (inset, ×400); **Right**, right posterior iliac bone marrow (hematoxylin and eosin, ×100) was filled with normal cells (inset, ×400). Immunohistochemical studies showed that CD43, TDT, CD99, and PAX 5 were positive in the left posterior iliac bone marrow cells (×400).

**Figure 3:**
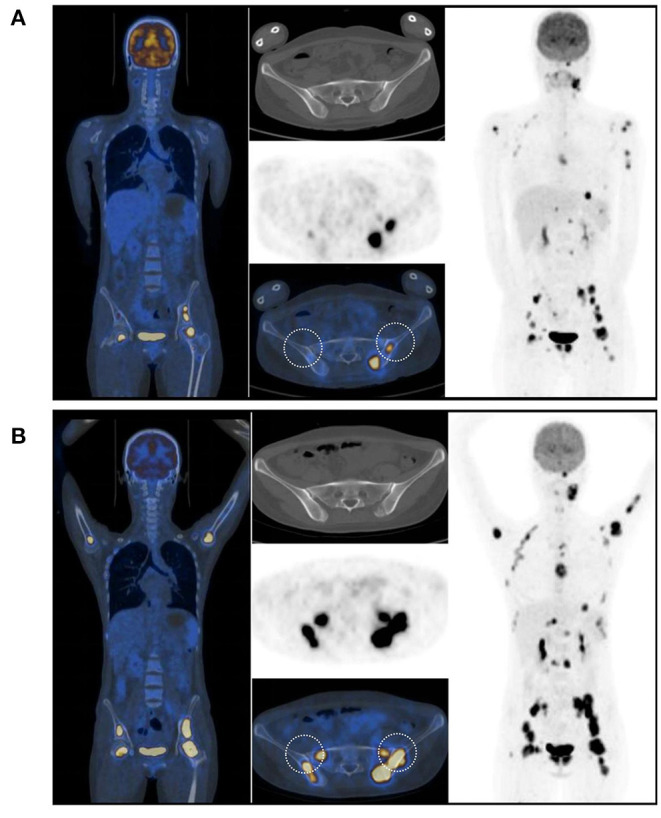
^18^F-FDG-PET/CT scans showed that asymmetrical hypermetabolic foci were noted across the bone marrow system. **(A)** on the day +143. **(B)** on the day +203. Both aspirations and biopsies were performed in the circle areas.

In July 2017, a 23-year-old woman was diagnosed with acute B lymphoblastic leukemia with t (4, 11) (q13; q21). Bone marrow aspirate smear from her right posterior iliac crest revealed the presence of 85.5% blasts, and reverse transcription polymerase chain reaction confirmed the presence of *MLL-AF4* fusion transcripts. After a cycle of induction chemotherapy, her bone marrow showed CR. Following two cycles of consolidation, she received G-CSF-mobilized PBSC from her HLA-identical sister on December 9–11, 2017. The conditioning regimen consisted of busulfan (3.2 mg/kg/day, −9 to −7), etoposide (10 mg/kg/day, −6 to −4) and cyclophosphamide (60 mg/kg/day, −3 and −2). Cyclosporine and short-term MTX were used to prevent GVHD. Her neutrophils and platelets engrafted on day +9 and +11, respectively. The bone marrow aspiration schedule is shown in Figure 1. On day +30, the bone marrow specimens from her left posterior iliac crest showed 3–9% recipient chimerism and low copy of *MLL-AF4* transcript (0.86%, %copies/*ABL*). The *MLL-AF4* copies almost increased 20 times to 18.87% on day +51. Oral cyclosporine was tapered quickly and discontinued on day +77, followed by stage 2 acute GVHD of skin. Biopsy of her right posterior iliac crest bone marrow showed two times full donor chimerism and the copy of *MLL-AF4* was decreased to 0.05% on day +100. Unfortunately, on day +122, up to 62% of blasts were found in her left posterior iliac crest bone marrow smear and no blast forms were seen in the peripheral blood smear. However, on day +128, the blast percentage was 0% in the right (Figure 1). On day +133, a bone marrow biopsy from bilateral posterior iliac crests showed that the left bone marrow was replaced with lymphoblasts and the right was normal (Figure 2). Immunohistochemical studies showed that the lymphoblasts expressed CD43, TDT, CD99 and PAX5, and did not express CD3, CD20, MPO, CD34, CyclinD1 and Ki67 (Figure 2). Facing this rare leukemia progression, the patient and her donor agreed to pursue chemotherapy combined with donor lymphocyte infusion (DLI) as soon as possible. In order to evaluate the distribution of the disease before treatment, fluorine-18-fluorodeoxyglucose positron emission tomography/computed tomography (^18^F-FDG-PET/CT) was performed on day +143. Unexpectedly, asymmetric metabolic abnormalities were found throughout the bone marrow system, and there was no corresponding anatomical change on CT imaging (Figure 3A). The maximal Standardized uptake value (SUV_max_) of left rambus mandibulae, humerus and ilium were up to 18.5, 18.4 and 21, respectively. Considering the higher leukemic burden in the left, we chose the left posterior iliac crest site for further response assessment. There was no apparent active GVHD, she achieved transient hematologic CR after chemotherapy combined with DLI. On day +203, subsequent ^18^F-FDG-PET/CT scan revealed similar metabolic abnormalities in the bone marrow system (Figure 3B). On day +229, an aspiration from bilateral posterior iliac crests showed that the blast percentage of the left bone marrow smear was 62% and the right was 3.5%. Unfortunately, she died of septic shock with heart failure on day +258.

## Discussion

Given the well-known localized infiltrating characteristics, chronic lymphoid leukemia, lymphoma, multiple myeloma, and neuroblastoma, bilateral bone marrow aspiration and/or biopsies are usually performed for staging. Acute leukemia is characterized by a uniform bone marrow infiltration of leukemic cells (6) so bone marrow aspirations in acute leukemia patients are always performed on a unilateral iliac crest post-HSCT. Compared with classical morphology enumeration methods, minimal residual disease (MRD) detecting tools such as multi-parameter flow cytometry (MFC), cytogenetics or molecular studies are far more sensitive to detect leukemic blasts. Tetsuo Maeda and colleagues reported a case that presented as an apparent discrepancy in the DEK-CAN fusion transcript levels between the left and right iliac bone marrow sites during hematological CR, and attributed it to anatomic differences in the potency of the GVL response of DLI (7). As shown in the Table 1, for our patient, unlike the left ilium where a high burden of leukemic cells was detected, the immunophenotype, cytogenetics and recipient chimerism of the right ilium were completely negative. The patient had been off immunosuppression agents for more than a year with no symptoms of GVHD, and he refused all therapies during the three bone marrow aspirations in a month. Although we did not perform concurrent bilateral bone marrow aspirations/biopsies, case 1 reminds us that patients without immune antitumor effects may develop different leukemia reservoirs in the bone marrow system.

Because MRD was detected early after allo-HSCT, cyclosporine was rapidly reduced and discontinued on day +77 in case 2. On day +100, the active GVL effect appeared to significantly control the MRD of her right posterior iliac bone marrow. Due to repeated right posterior iliac crest aspirations, completely wrong conclusions were almost drawn. On day +122, the blast percentage of her left posterior iliac crest bone smear had increased to 62%, while on the day +128, the right still showed CR. A subsequent bone marrow biopsy from bilateral posterior iliac crests revealed that blasts almost completely infiltrated only in the left. This case reminds us that unilateral/single-site bone marrow aspiration may have deficiencies in the detection of residual disease or the evaluation of treatment effects. However, the number of cases is too small to draw convincing conclusions.

Two whole-body ^18^F-FDG-PET/CT scans showed that hypermetabolism in the bone marrow system was significantly asymmetric. There is no consensus on the diagnostic and predictive value of PET/CT for intramedullary acute leukemia. Several papers reported that PET/CT incidentally detected acute lymphoblastic leukemia (ALL) (8–11), and a few studies have reported the evaluation and predictive value of PET/CT in patients with leukemia (12, 13). The prospective PETAML trial reported that the specificity of ^18^F-FDG-PET/CT for diagnosis of extramedullary acute myeloid leukemia was 97%, despite hematological remission (14). In a retrospective study of 28 children suspected of leukemia progression or recurrence during / after chemotherapy or allo-HSCT, 14 cases with positive ^18^F-FDG-PET/CT scans were associated with increased blasts in bone marrow biopsies, and the mean SUV_max_ was significantly higher than what is seen with infectious diseases (15). In addition, G-CSF induced high uptake of FDG in bone marrow system was always diffusely distributed (16, 17). In the second case, the adjacent bilateral bone biopsy (day +133) and PET/CT scan (day +143) demonstrated that the focal bone marrow hypermetabolism of ^18^F-FDG was caused by the asymmetric distribution of the blasts.

In 1972, a 3$\frac{1}{2}$ year-old-boy was diagnosed with ALL in the St. Louis Children's Hospital. The bone marrow of both iliac crests was found to be replaced with lymphoblasts, however, a bone marrow aspirate from the spinous process of the first lumbar vertebra was almost normal (18). Such morphologic discordance reminds us that leukemia cells do not always evenly infiltrate through the bone marrow system. Endo, T. and colleagues occasionally found the first case of localized relapse of ALL in bone marrow extremities after allo-HSCT because of extremities pain (19). Golembe, B. and colleagues found discordant bone marrow specimens in an 11-year-old ALL patient who had been in complete remission for 6 years and off chemotherapy for 2$\frac{1}{2}$ years. Three months later, bone marrow samples of three sites showed leukemic involvement (20). The final hematology relapse of the case and our case 1 indicates that there may be a precursor state of relapse in focal bone marrow sites before general relapse. To the best of our knowledge, with the combination of bilateral bone marrow aspiration/biopsy and whole body ^18^F-FDG-PET/CT, our case report first illustrates the asymmetric bone-marrow infiltration of leukemic cells after allo-HSCT. Bone marrow aspirations were performed more frequently than usual, which may be why these incredible results were observed. However, the underlying mechanism and the exact interval between the asymmetric bone marrow recurrence and the subsequent systemic bone marrow relapse need to be confirmed by further studies.

## Conclusions

Because the number of cases is still too small, it is not appropriate to perform bilateral posterior iliac crest aspiration or ^18^F-FDG-PET/CT scan for every acute leukemia patient after allo-HSCT. However, if discordant bone marrow specimens are observed, providers need to consider these rare cases in addition to the quality control issues of bone marrow aspiration. ^18^F-FDG-PET/CT scans or bilateral posterior iliac crest aspirations may help distinguish the asymmetric bone marrow distribution of blasts, and then further aspirations should be conducted on the side with more blasts to avoid inaccurate efficacy assessments.

## Data Availability Statement

The original contributions presented in the study are included in the article/Supplementary Material, further inquiries can be directed to the corresponding author/s.

## Ethics Statement

The work has been evaluated and approved by the ethical committee of Tongji Medical College, Huazhong University of Science and Technology. The patients/participants provided their written informed consent to participate in this study. Written informed consent was obtained from the individual(s) for the publication of any potentially identifiable images or data included in this article.

## Author Contributions

HY, WS, YW, ZZhon, and YY obtained and analyzed the clinical data. ZZhou and XX made the figures. JY and LX performed the MFC and RT-PCR. WC provided morphology analysis. All authors contributed to caring for the patient, editing the figures, and writing and editing the manuscript.

## Conflict of Interest

The authors declare that the research was conducted in the absence of any commercial or financial relationships that could be construed as a potential conflict of interest.

## Abbreviations

Allo-HSCT: allogeneic hematopoietic stem cell transplantation
GVL: graft vs. leukemia
CR: complete remission
G-CSF: granulocyte-colony stimulating factor
PBSC: peripheral blood stem cell
HLA: human leukocyte antigen
MTX: methotrexate
GVHD: graft vs. host disease
DLI: donor lymphocyte infusion
18F-FDG-PET/CT: fluorine-18-fluorodeoxyglucose positron emission tomography/computed tomography
SUVmax: maximal Standardized uptake value
MRD: minimal residual disease
MFC: multi-parameter flow cytometry
ALL: acute lymphoblastic leukemia.
