# Supplementary figures and images for: Case Report: Asymmetric Bone Marrow Involvement in Patients With Acute Leukemia After Allogeneic Hematopoietic Stem Cell Transplantation
Source: Front Oncol. 2021 Mar 4;11:626018. doi: 10.3389/fonc.2021.626018 (PMC7970045; doi:10.3389/fonc.2021.626018)

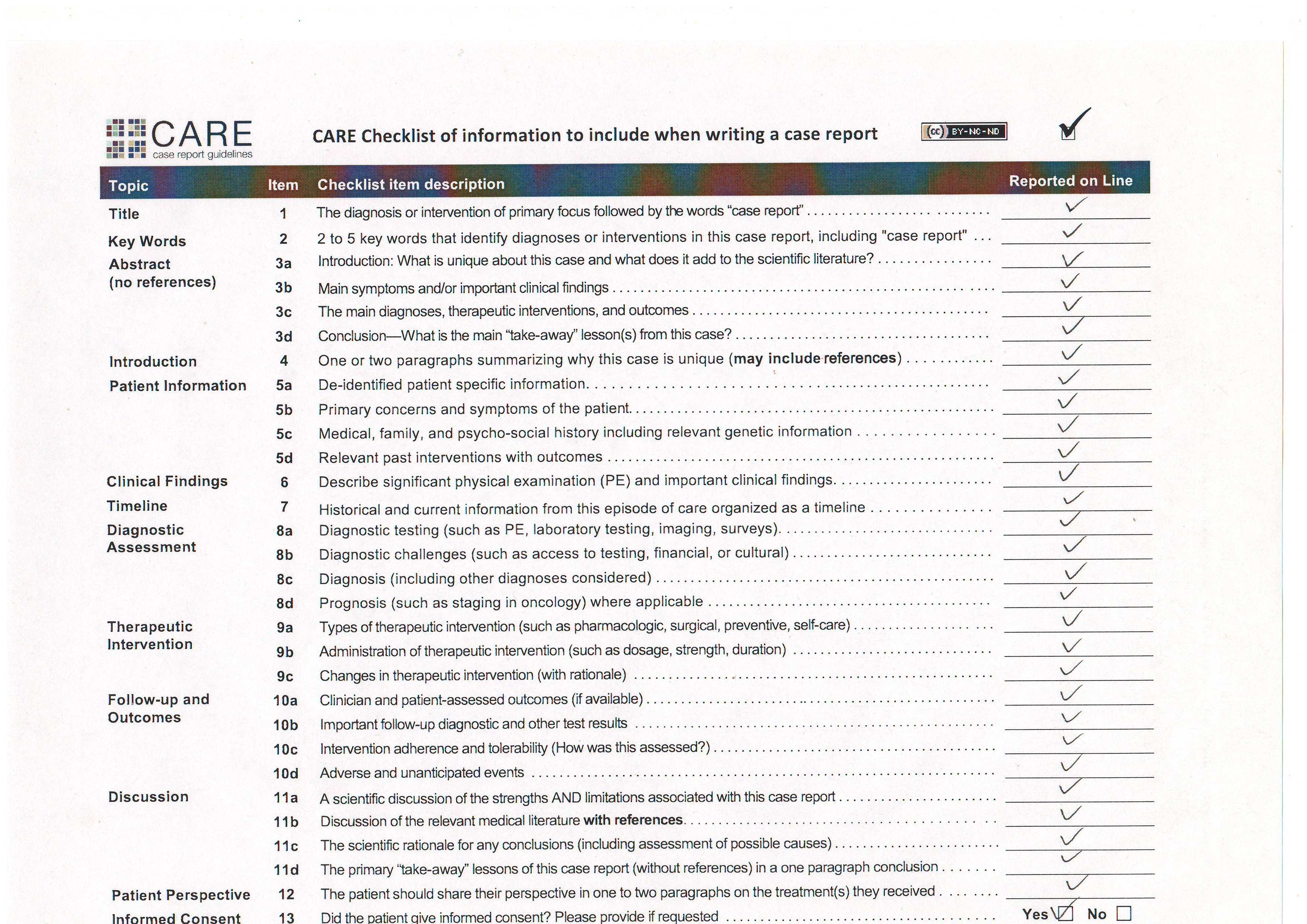

Supplement: Supplementary file 1 [file Image_1.JPEG]
